# Loss of Somatostatin Receptor Subtype 2 in Prostate Cancer Is Linked to an Aggressive Cancer Phenotype, High Tumor Cell Proliferation and Predicts Early Metastatic and Biochemical Relapse

**DOI:** 10.1371/journal.pone.0100469

**Published:** 2014-07-10

**Authors:** Jan K. Hennigs, Julia Müller, Matti Adam, Joshua M. Spin, Emilia Riedel, Markus Graefen, Carsten Bokemeyer, Guido Sauter, Hartwig Huland, Thorsten Schlomm, Sarah Minner

**Affiliations:** 1 Department of Pathology, University Medical Center Hamburg-Eppendorf, Hamburg, Germany; 2 Department of Internal Medicine II - Oncology, Hematology, BMT with Section Pneumology, Hubertus-Wald-Tumorzentrum/University Cancer Center Hamburg (UCCH) University Medical Center Hamburg-Eppendorf, Hamburg, Germany; 3 Division of Cardiovascular Medicine and Cardiovascular Institute, Stanford University – School of Medicine, Stanford, California, United States of America; 4 Martini Clinic, Prostate Cancer Center, University Medical Center Hamburg-Eppendorf, Hamburg, Germany; University of Salerno, Faculty of Medicine and Surgery, Italy

## Abstract

Somatostatin receptor subtype 2 (SSTR2) is the most frequently expressed SSTR subtype in normal human tissues. SSTR2 expression is differentially regulated in various tumor types and therapeutic somatostatin analogs binding to SSTR2 are in clinical use. In prostate cancers highly contradictory results in terms of SSTR2 expression and its consequences have been published over the past years. The aim of this study was to clarify prevalence and clinical significance of SSTR2 expression in prostate cancer. Therefore, quantitative immunohistochemistry (IHC) using a tissue microarray containing samples from 3,261 prostate cancer patients with extensive clinical and molecular cancer characteristics and oncological follow-up data was performed. IHC data was compared to publicly available Gene Expression Omnibus datasets of human prostate cancer gene expression arrays. While membranous SSTR2 staining was always seen in normal prostate epithelium, SSTR2 staining was absent in more than half (56.1%) of 2,195 interpretable prostate cancer samples. About 13% of all analyzed prostate cancers showed moderate to strong cytoplasmic and membranous SSTR2 staining. Staining intensities were inversely correlated with high Gleason grade, advanced pT category, high tumor cell proliferation (p<0.0001 each), high pre-operative PSA levels, (p = 0.0011) and positive surgical margins (p = 0.006). *In silico* analysis confirmed lower SSTR2 gene expression in prostate cancers vs. normal adjacent tissue (p = 0.0424), prostate cancer metastases vs. primary cancers (p = 0.0011) and recurrent vs. non-recurrent prostate cancers (p = 0.0438). PSA-free survival gradually declined with SSTR2 staining intensity (p<0.0001). SSTR2-negative cancers were more likely to develop metastases over time (p<0.05). In conclusion, most prostate cancers are indeed SSTR2-negative and loss of SSTR2 strongly predicts an unfavorable tumor phenotype and poor prognosis. Therefore, SSTR2 expression seems an important factor in the pathogenesis of prostate cancer and re-introduction of the receptor in SSTR2-negative prostate cancers may feature a promising target for novel gene therapy approaches.

## Introduction

In Western males, prostate cancer is the most frequently diagnosed malignant tumor with the second highest rate of cancer-attributed death [1]. Prostate cancers feature considerably variable courses of disease from slow local growth to aggressive and invasive metastatic proliferation. Therefore, a thorough molecular characterization of different prostate cancer subtypes is essential in order to discriminate between aggressive and non-aggressive cancer phenotypes.

Somatostatin (SST) is a cyclic neuroendocrine hormone which was originally isolated from sheep hypothalamus as *in vitro* inhibitor of growth hormone action (GH, [2]). SST is produced by neuroendocrine cells throughout the whole body and is therefore widely expressed. This comprises the central and peripheral nervous system and a variety of cells within the digestive, genitourinary and reproductive tracts [3].

SST exists in two biologically active isoforms (SST-14 and SST-28) which bind to 5 distinct membrane surface receptors (SSTR1-5) with variable binding affinities [4]. Upon activation by SST-14/-28 all SSTRs inhibit the generation of cyclo-AMP thereby diminishing mitogen-activated (MAP) kinase mediated cell proliferation in a broad variety of cell types [3]. The most widely expressed SSTR subtype in normal tissues [3] is SSTR2.

Various synthetic SSTR agonists are available, of which the anti-proliferative octapeptide octreotide is the most popular representative that is now routinely used for detection and therapy of neuroendocrine tumors [5]. In addition, by agonist profiling octreotide was identified as most potent binding partner of SSTR subtype 2 (SSTR2, [4]).

Using labeled octreotide binding assays, in situ hybridization, immunohistochemistry and RNA-binding assays SSTR2 expression has been shown to be also expressed in a variety of malignant and non-malignant tumors such as pituitary adenoma, meningeoma, neuroblastoma, Non-Hodgkin’s lymphoma, neuroendocrine carcinoid tumors, breast tumors, renal, pancreatic and small cell lung cancers [3]. In these cancers SSTR2 activation leads to an inhibition of tumor cell proliferation, mostly mediated via growth arrests [3].

In prostate cancer there are multiple contradictory reports regarding the role and expression of SSTR2 (for a summary see [6]): Hansson et al., for instance, suggest an up-regulation of SSTR2 expression in prostate cancers [7], Morichetti and coworkers found increased SSTR2 immunostaining in 80% of incidental prostate cancers [8]. Other groups found diminished or absent SSTR2 expression in prostate cancers [9], [10].

In the light of these conflicting reports, we analyzed SSTR2 expression by immunohistochemistry on a large (3,261 tumors) prostate cancer tissue microarray (TMA) and by publicly available Gene Expression Omnibus (GEO) datasets of human prostate cancer gene expression arrays in order to comprehensively clarify prevalence and clinical significance of SSTR2 expression in prostate cancers.

## Materials and Methods

### Tissue microarray construction

The prostate cancer prognosis tissue microarray (TMA) consisted of cancer samples from 3,261 patients distributed over 7 paraffin blocks. The sampling and constructions have been previously described in detail [11]. In brief, specimens from radical prostatectomies performed between 1992 and 2005 at the Department of Urology, University Medical Center Hamburg-Eppendorf were paraffin-embedded and afterwards matched with clinico-pathological data. TNM classification (American Joint Committee on Cancer, 2002, 6^th^ edition) was used for tumor staging in order to define primary tumor size and local invasiveness (pT), regional lymph node affection (pN) and distant spreading/metastatic disease (pM). Grading of cancers was performed using the Modified Gleason Score [12] and by evaluation of cancer-free surgical margins [13]. In all patients undergoing radical prostatectomy, prostate specific antigen (PSA, [14]) concentrations were measured at the time of diagnosis and for post-operative surveillance quarterly in the first year followed by biannual measurements in the second and annual measurements after the third year following surgery. Recurrence was defined as a postoperative PSA of 0.2 ng/ml. Time of recurrence was defined by the first PSA value above or equal to 0.2 ng/ml. Patients without evidence of tumor recurrence were censored at last follow-up. No patient of the cohort received neo-adjuvant or adjuvant therapy. For TMA construction, representative tissue cylinders with a diameter of 600 µm were punched from tumor areas of a paraffin-embedded donor tissue block and transferred to the corresponding coordinates on the recipient paraffin block in a half-automated process using precision instruments. Four-micrometer thick sections of each microarray block were transferred to adhesive slides for immunohistochemistry analyses.

Use of tissues and clinical data was approved by the ethics committee of the Hamburg Chamber of Physicians and in accordance with local law (Hamburgisches Krankenhausgesetz, HmbKHG) and the Declaration of Helsinki. In agreement with HmbKG, §12, 1–3 and §12a, 1–5 specific informed consent was neither required nor obtained for the present study. All patient records/information were anonymized and de-identified prior to analyses.

### Immunohistochemistry (IHC)

Freshly cut TMA sections were stained in one experiment on a single day. TMA sections were de-paraffinized followed by heat-induced antigen retrieval in an autoclave in acetate buffer pH 6.0 for 5 min. Primary polyclonal rabbit anti-SSTR2 antibody (HPA007264, Atlas Antibodies, Stockholm, Sweden) was used in a final dilution of 1∶150. SSTR2 expression was visualized utilizing the Envision System (DAKO, Glostrup, Denmark).

Membranous and cytoplasmatic staining was evaluated separately for each spot. The staining intensity (negative = 0, weak = 1+, intermediate = 2+, strong = 3+) and the fraction of positive tumor cells (in %) were recorded for each tissue spot. A final score was built from these two parameters as previously described [15], [16]. In brief, negative scores had staining intensity of 0, weak scores had staining intensity of 1+ in ≤70% of tumor cells or staining intensity of 2+ in ≤30% of tumor cells; moderate scores had staining intensity of 1+ in >70% of tumor cells, staining intensity of 2+ in >30% and ≤70% of tumor cells or staining intensity of 3+ in ≤30% of tumor cells and strong scores had staining intensity of 2+ in >70% of tumor cells or staining intensity of 3+ in >30% of tumor cells. Ki67 IHC data generated on the same TMA were available from a previous study [17].

### In silico cDNA microarray analysis

A Gene Expression Omnibus (GEO) search (www.ncbi.nlm.nih.gov/gds) was conducted for human gene array datasets with information on SSTR2 expression using the string “prostate cancer somatostatin receptor 2 homo sapiens”. Additional requirements were: expression data within the same dataset for comparison of (1) healthy prostate and prostate cancer, (2) primary prostate cancers and metastatic prostate cancers, or (3) non-recurrent and recurrent prostate cancers. Furthermore, suitable datasets needed to include a minimum of n = 22 per group in order to accomplish a Type I error probability of 0.05 to reach statistical power of 0.9 and a difference in groups of one standard deviation in a non-paired sample setting.

Out of a total of 158 datasets identified with expression data on SSTR2 in human prostate cancers only 2 datasets fulfilled all of the criteria above, namely GDS2545 [18], [19] and GDS4109 [20].

GDS2545 contains expression data from 65 primary prostate cancers, 63 normal tissues adjacent to prostate cancer and 25 prostate cancer metastases hybridized to the Affymetrix Human Genome U95 Version 2 Array platform (GPL8300). GDS4109 contains expression data from 39 recurrent and 49 non-recurrent primary prostate cancers hybridized to the Affymetrix Human Genome U133A Array platform (GPL96).

Normalized gene expression values for SSTR2 (GDS2545+ GDS4109) as well as SSTR3, 4 and 5 (GDS2545 only) were extracted and analyzed using the GEO Dataset Browser data analysis online tools. No information was available for SSTR1 from GDS2545.

### Statistical analysis

Statistical analyses were accomplished using JMP 5.0.1 software (SAS Institute Inc., Cary, NC, USA) performing Pearson’s chi-square test for contingency tables. Analysis of Variances (ANOVA) was used to test the association of Ki67 labeling index (LI) and SSTR2 staining, Kolmogorov-Smirnov test for determination of normal distribution and subsequent unpaired t-test or Mann-Whitney-U test calculations were performed for *in silico* cDNA expression analysis using PRISM 6 (Graphpad Software, Inc, La Jolla, CA, USA). Survival curves were calculated by Kaplan–Meier analysis and compared with log rank test. Multivariate analysis utilized the Cox regression model to identify independence of clinical parameters and SSTR2 immunohistochemistry to predict PSA-recurrence, cancer-specific survival and time to onset of metastatic disease after radical prostatectomy.

## Results

### Baseline characteristics of prostate cancer patients

A TMA was constructed from cancer tissues after radical prostatectomies from 3,261 patients treated at the Department of Urology, University Medical Center Hamburg-Eppendorf between 1992 and 2005. Follow-up data for biochemical cancer relapse were available for 2,385 patients (73.1%) with a mean observation period of 34.7 months (range: 1 to 143 months, see Table 1 for baseline characteristics). TMA cores of 2,363 patients contained prostate cancer cells (72.5%). These cores were included in this study. The group of interpretable cases for statistical analysis was formed by 2,195 spots (67.3%) with immunohistochemical information regarding SSTR2 expression.

**Table 1:** Baseline Characteristics of 2,363 prostate cancer patients included in the IHC study*.

Parameter	Variable	n =	%
Age at diagnosis [y]	<50	57	2.6
	50–60	701	31.8
	60–70	1,317	59.7
	>70	131	5.9
Pre-operative PSA [ng/ml]	<4	339	15.4
	4–10	1,178	53.5
	10–20	493	22.4
	>20	191	8.7
Gleason score	≤3+3	932	41.6
	3+4	1,002	44.7
	4+3	257	11.5
	≥4+4	49	2.2
pT category (AJCC 2002)	pT2	1,405	62.8
	pT3a	495	22.1
	pT3b	305	13.6
	pT4	31	1.5
pN category	pN0	1,150	51.5
	pN+	79	3.5
	pNx	1,005	45.0
Surgical margins	Negative	1,749	78.2
	Positive	488	21.8
Total:		2,363*	

*Deviations from total are due to missing data in the subcategories.

(AJCC = American Joint Committee on Cancer, PSA = Prostate-specific antigen).

### Loss of SSTR2 expression in prostate cancer is strongly linked with biologically aggressive cancers

**Figure 1:**
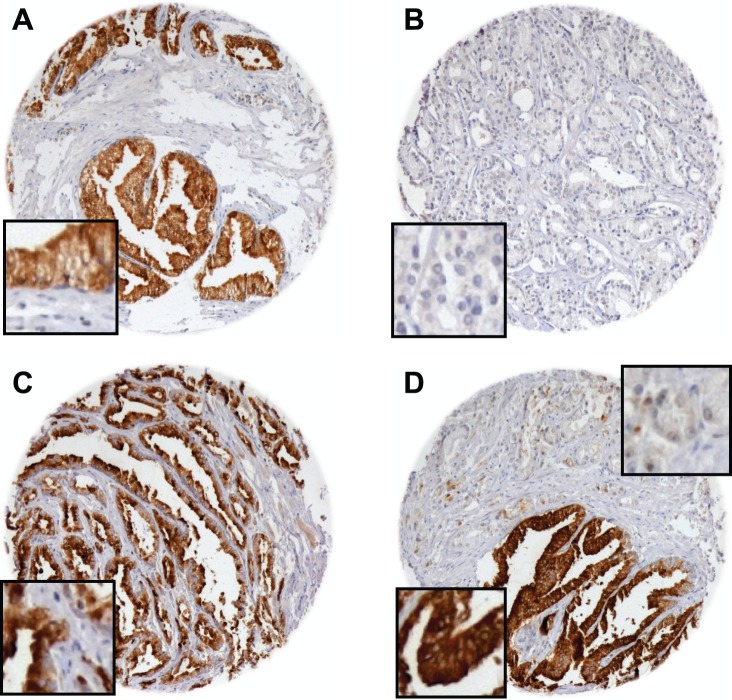
SSTR2 immunohistochemistry in normal prostate tissue and prostate cancer. Microphotographs of tissue microarray cores showing normal prostate and prostate cancer tissues: SSTR2-positive normal epithelium (**A**), SSTR2-negative (**B**) and SSTR2-positive prostate cancer tissue (**C**) as well as SSTR2-negative cancer cells next to strongly SSTR2-positive normal epithelial cells (**D**).

SSTR2 expression as determined by IHC was absent in 1,231 (56.1%) of interpretable prostate cancer samples. Weak SSTR2 staining was detected in 680 of these tumors (31.0%). Moderate and strong staining occurred in 187 (8.5%) and 97 prostate cancers (4.4%), respectively. Staining pattern was mostly both membranous and cytoplasmic throughout positive TMA spots. For comparison, normal prostate tissue always showed a positive receptor staining (Figure 1A–D).

Contingency analysis of tumor phenotype and clinical features revealed significant, inverse associations of SSTR2 staining intensity with de-differentiation of tumors (as indicated by high Gleason grade, p<0.0001), advanced tumor stage of prostate cancers (as indicated by advanced pT category, p<0.0001, and cancer-positive surgical margins, p = 0.006) as well as high pre-operative PSA levels (p = 0.0011). Statistical details on the correlation of clinico-pathological factors with SSTR2 IHC intensities and frequencies are given in Table 2.

**Table 2:** SSTR2 IHC intensities and frequencies in patients with radical prostatectomy.

Parameter	Variable	n =	negative (%)	weak (%)	moderate (%)	strong (%)	p-value
Pre-operative PSA[ng/ml]	<4	304	53.0	33.2	9.2	4.6	0.0011
	4–10	1,091	53.5	31.5	9.8	5.1	
	10–20	462	58.4	31.0	6.9	3.7	
	>20	183	71.6	21.3	4.9	2.2	
Gleason score	≤3+3	858	51.5	31.9	10.4	6.2	<0.0001
	3+4	930	55.4	32.4	8.3	4.0	
	4+3	243	71.6	23.5	4.1	0.8	
	≥4+4	46	78.3	17.4	4.4	0.0	
pT stage	pT2	1,277	51.5	32.0	11.0	5.6	<0.0001
	pT3a	470	63.0	28.3	5.5	3.2	
	pT3b	297	63.3	31.0	4.0	1.7	
	pT4	32	78.1	21.9	0.0	0.0	
pN stage	pN0	1,093	57.9	32.3	6.2	3.6	0.0963
	pN+	76	65.8	29.0	5.2	0.0	
Surgical MarginStatus	Negative	1,613	54.3	32.0	9.2	4.5	0.0060
	Positive	461	62.9	26.9	6.1	4.1	

Deviations from total are due to missing data in the subcategories and rounding.

**Figure 2:**
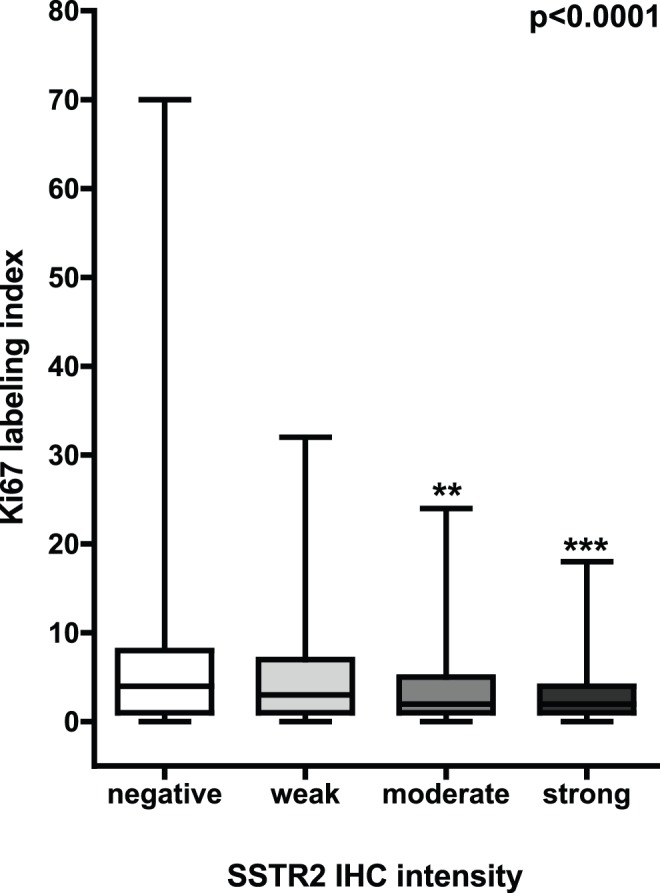
Ki67 labeling index and SSTR2 immunohistochemistry (IHC) in prostate cancer cells. Ki67 labeling index shows a strong inverse correlation with SSTR2 staining intensities (p<0.0001, ANOVA, Dunn’s multiple comparison post-hoc test, box-and-whiskers graph plotting median, 25^th^ and 75^th^ percentile, ** = p<0.01 vs. negative SSTR2 IHC, *** = p<0.001 vs. negative SSTR2 IHC).

The same strong inverse correlation with SSTR2 staining intensity was detected with tumor cell proliferation as determined by Ki67 LI. Ki67 LI increased with decreasing SSTR2 IHC intensity in prostate cancer cells from 2.8±0.4 (strong SSTR2 IHC) over 3.8±0.4 (intermediate SSTR2 IHC) and 5.0±0.2 (weak SSTR2 IHC) to 5.9±0.2 in SSTR2-negative prostate cancers (arithmetical mean ± standard error of the mean, p<0.0001, Figure 2).

To test if the changes in SSTR2 expression detected by IHC could be confirmed on the transcriptional level, we performed *in silico* expression analysis utilizing two publicly available GEO datasets of human prostate cancer gene expression arrays (GDS2545 and GDS4109).

**Figure 3:**
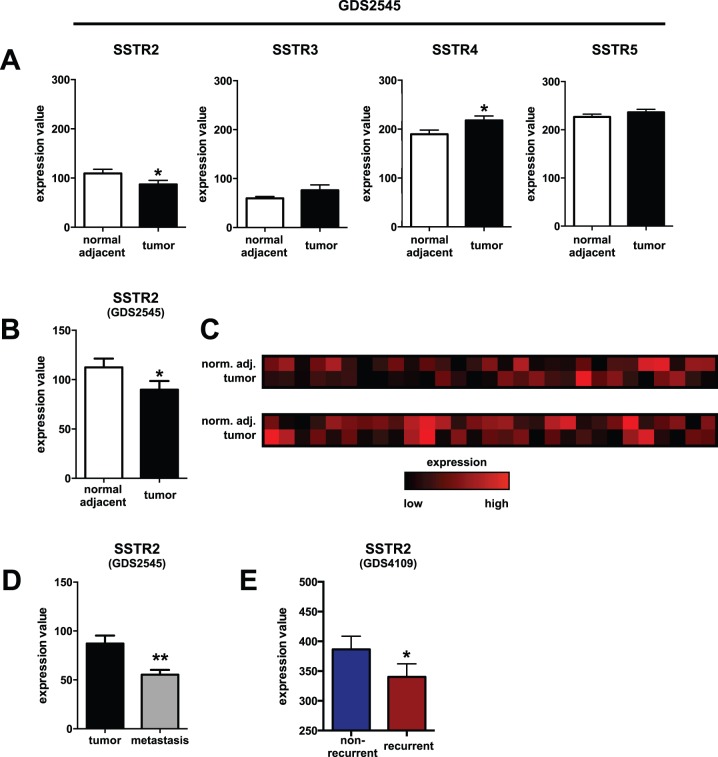
In silico analysis of SSTR gene expression in prostate cancers. Gene expression analysis of SSTR subtypes in primary prostate cancer tissues versus normal adjacent prostate tissues shows increased SSTR4 (p = 0.0236) and confirms lower SSTR2 expression (p = 0.0424) in primary prostate cancers when comparing all available samples with each other (**A**, n = 65 vs. 63 patients) or corresponding samples from the same patients only (**B**, n = 58). Levels of SSTR3 and SSTR5 expression are unchanged (p>0.05). A heatmap of relative SSTR2 gene expression in prostate cancer tissue (tumor) and the surrounding adjacent normal prostate (norm. adj.) per individual patient (column) is shown in (**C**). SSTR2 expression is also lower in prostate cancer metastases vs. primary tumor (**D**, p = 0.0011, n = 25) and in recurrent compared with non-recurrent prostate cancers (**E**, p = 0.0438, n = 39 vs. 40 patients). Standardized expression values were extracted from the identified GEO datasets GDS2545 [18], [19] and GDS4109 [20] and compared as described in the Methods section (Data given as Mean ± SEM; * = p<0.05 vs. corresponding control, ** = p<0.01 vs. corresponding control).

Using the GDS2545 dataset, we compared expression of SSTR subtypes 2, 3, 4 and 5 in normal prostate tissue with physically adjacent primary prostate cancer in an unpaired fashion. While expression of SSTR3 (mean normalized expression value ± SD: 59.7±30.1 [normal] vs. 76.2±89.1 [cancer], p>0.05) and SSTR5 (226.3±47.1 [normal] vs. 236.1±50.6 [cancer], p>0.05) did not differ significantly, SSTR4 expression was increased (189.7±65.8 [normal] vs. 217.9±73.1 [cancer], p = 0.0236) and SSTR2 expression was significantly lower in prostate cancers (109.5±67.4 [normal] vs. 87.2±65.3 [cancer], p = 0.0424, Figure 3A).

This was also the case when comparing SSTR2 expression in prostate cancer with the corresponding normal adjacent tissue from the same patient using paired analysis (112.5±67.9 [normal] vs. 89.8±66.9 [cancer], p = 0.0486, n = 58 patients, Figure 3B). In total, prostate cancers from 36 patients (62.1%) showed lower SSTR2 expression than their corresponding normal adjacent tissue (Figure 3C). Prostate cancer metastases had even lower SSTR2 expression values than primary prostate cancers (109.5±67.4 [primaries] vs. 55.4±23.9 [metastases], p = 0.0011, Figure 3D). In addition, using the GDS4109 dataset, recurrent prostate cancer showed lower SSTR2 expression compared to non-recurrent cancers (386.5±140.5 [non-recurrent] vs. 340.2±137.0 [recurrent], p = 0.0438, Figure 3E).

### Loss of SSTR2 in prostate cancers predicts metastatic and biochemical cancer relapse

**Figure 4:**
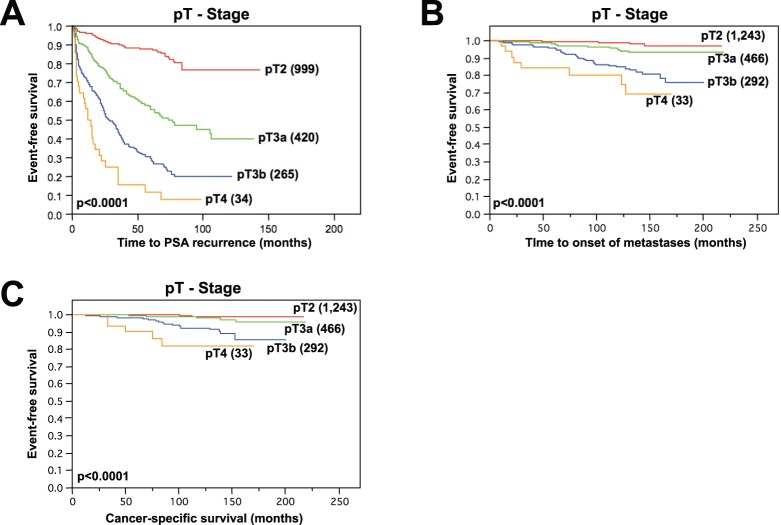
pT stage and event-free survival in prostate cancer patients. Kaplan-Meier curves showing pT stage dependence of PSA-recurrence free survival (**A**), metastasis-free survival (**B**) and cancer-specific survival (**C**, all p<0.0001, Log-Rank test) in radically prostatectomized prostate cancer patients.

In Kaplan-Meier analysis, all tested clinical and pathological features were strongly linked to PSA recurrence, prostate cancer specific survival and time to onset of metastatic disease. This applied for Gleason score, pT stage, pN Stage, pre-operative PSA levels and surgical margin status (all p<0.0001, Table 3 and Figure 4A–C).

**Table 3:** Association of clinico-pathological features of prostate cancer samples included in this study with PSA recurrence, cancer-specific survival and time to onset of metastatic disease.

	Log-Rank test *(Kaplan-Meier analysis)*					
Parameters	PSA recurrence		Cancer-specific survival		Metastasis-free survival	
	χ^2^	P value	χ^2^	P value	χ^2^	P value
Pre-operative PSA [ng/ml]	111.0	<0.0001	10.4	0.0157	20.7	0.0001
						
Gleason score	425.3	<0.0001	96.1	<0.0001	166.1	<0.0001
						
pT category (AJCC 2002)	418.0	<0.0001	63.2	<0.0001	103.8	<0.0001
						
pN category	182.6	<0.0001	9.2	0.0024	25.5	<0.0001
						
Surgical margin status	111.7	<0.0001	28.1	<0.0001	31.4	<0.0001

**Figure 5:**
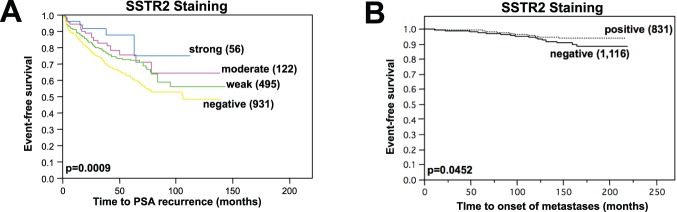
Clinical impact of SSTR2 staining on event-free survival. PSA recurrence-free survival gradually declines from strong staining of cancer spots over moderate and weak to SSTR2-negative prostate cancers (**A**, p = 0.0009, Kaplan-Meier analysis with Log-Rank test). Prostatectomized patients with SSTR2-negative prostate cancers also have impaired metastasis-free survival (**B**, p = 0.0452, Kaplan-Meier analysis with Log-Rank test).

Except for prostate cancer-specific survival (p = 0.5942, not shown) SSTR2 staining was inversely correlated with biochemical recurrence and metastasis-free survival: Respectively, PSA free survival (p = 0.0009, Figure 5A) and metastasis-free survival (p = 0.0452, Figure 5B) gradually declined from strong SSTR2 staining over medium and weak to negative.

In multivariate analyses, all tested parameters (Gleason score, pT stage, pN Stage, surgical margin status and pre-operative PSA levels, all p<0.001) but not SSTR2 staining intensity (p = 0.6938) were identified as independent risk factors for biochemical relapse. Comparable results were found for metastasis-free survival (SSTR2 staining, p = 0.8443).

## Discussion

Immunohistochemically detectable cytoplasmic and membranous SSTR2 protein was seen in 44% of our 2,195 interpretable prostate cancer samples. Previous studies had analyzed smaller patient cohorts (14–45 cases) and found highly variable results including lower [9], [10] and higher [7], [8] numbers of “SSTR2-positive” prostate cancers as compared to our data. By RT-PCR, Halmos et al. only found three SSTR2-positive prostate cancers out of 22 samples [9]. Cariaga-Martinez et al. report diminished or absent SSTR2 expression in 40 out of 45 prostate cancers using immunohistochemistry [10]. Hansson et al. suggested an up-regulation of SSTR2 expression in 12 out of 14 prostate cancer samples based on RNA in-situ hybridization [7]. Morichetti et al. recently reported a weak to intermediate cytoplasmic SSTR2 IHC staining in approx. 80% of prostate cancers from radical prostatectomies [8].

The comparison of normal and cancerous prostate epithelium in the present study by IHC demonstrated that SSTR2 is not overexpressed, but instead generally downregulated in prostate cancers. Our IHC data were confirmed by two independent cDNA microrarray datasets evaluated for SSTR2 gene expression. Furthermore, our findings are also supported by early data from studies using *in vitro* receptor autoradiography to compare SSTR2 expression between normal and neoplastic prostate epithelium [21]. Our data show, that SSTR2 downregulation is strongly linked to unfavorable tumor phenotype, early PSA relapse and onset of metastatic disease. This observation is also consistent with a previous study showing IHC positivity in 100% of 12 cases with Gleason grade 1 or 2 but in only 20% of 20 Gleason 4 or 5 cancers [10].

The strong association between high Ki67 LI and downregulated SSTR2 protein found in our study implies that SSTR2 downregulation exerts unfavorable biologic effects upon prostate epithelial cells through diminished cell proliferation control. Several studies have indeed suggested a role of SSTR2 in the regulation of tumor cell proliferation in various tumors, since tumors with reduced SSTR2 protein levels revealed increased cell proliferation [22]–[29]. For example, Qui et al. found significantly higher Ki67 LI in colorectal cancer cells with absent SSTR2 [25]. Over-expression of SSTR2 in MCF-7 breast cancer cells, which naturally express low levels of SSTR2, led to increased apoptosis and cell cycle arrest [29]. In C6 glioma cells, proliferation was inhibited by activation of SSTR2 as measured by [^3^H]thymidine incorporation assays [28], [30]. Additionally, infection of pancreatic and non-small cell lung cancer cells with SSTR2 expressing adenoviral vectors significantly decreased tumor growth and proliferation rate [27].

The mechanism, by which SSTR2 is able to confer its anti-proliferative effects, has recently been investigated. Zou et al. found a strong inhibitory effect of SSTR2 on the cell cycle in the aforementioned pancreatic cancer cells [27]. In their animal model for pancreatic adenocarcinoma, SSTR2 overexpression led to strong up-regulation of cyclin-dependent kinase inhibitor p16, which then inhibited tumor cell cycle progression from G1 to S phase. Recent data suggest that methylation may be a relevant mechanism for controlling SSTR2 expression in cancer. Torrisani et al. reported the regulation of human SSTR2 expression in various (cancer) cell lines by epigenetic modifications [31]. Their data showed that DNA methylation and histone acetylation can regulate the activity of the SSTR2 promoter and demonstrated that promoter activity directly correlates with SSTR2 expression in breast cancer, pancreatic cancer, hepatic cancer, melanoma and retinoblastoma cell lines in an inverse fashion. Moreover, treatment of these cells with demethylating agents and acetylase inhibitors rescued SSTR2 mRNA expression [31].

As a surface membrane receptor, SSTR2 is suitable as a therapeutic target. Since somatostatin’s short half-life limits its therapeutic use, synthetic analogs have been developed since the 1980s [32]. Octreotide is the best-characterized analog, and it binds to SSTR2 with higher affinity and much higher therapeutic potency than somatostatin [32]. Several other therapeutic ligands of SSTR2 are currently available or under clinical testing, including long-acting formulations (lancreotide, vapreotide, seglitide and pasireotide/SOM230) [33], and chimeric molecules coupled with cytotoxic agents (e.g. AN-238, a doxorubicin/somatostatin conjugate, [34]).

Octreotide and its derivatives have been used routinely for the detection and therapy of neuroendocrine tumors for years [35]. In C6 glioma, activation of SSTR2 by various somatostatin analogs led to a strong inhibition of *in vivo* cancer cell proliferation, intratumoral neoangiogenesis and Ki-67 expression [30].

Moreover, experimental treatment with the cytotoxic somatostatin analog AN-238 strongly inhibited tumor proliferation in a broad variety of SSTR2 positive cancer models such as Non-Hodgkin’s lymphoma [34], malignant melanoma [36], pheochromocytoma [37], endometrial [38], ovarian [39], colon [40] and gastric carcinomas [41] as well as small and non-small cell lung carcinoma [42], [43].

In concordance with our data, human pancreatic adenocarcinomas lose SSTR2 expression [44]. Re-introduction of SSTR2 in pancreas cancer by gene transfer robustly inhibited tumor cell proliferation and tumorigenicity [24], [45]. In the SSTR2-negative pancreatic and non-small lung cell cancer models of Zou and colleagues, re-expression of SSTR2 in the cancer cells led to significantly impaired tumor growth which was aggravated in a dose-dependent manner by application of octreotide and its derivate vapreotide (RC-160, [27]).

Lastly, we show that loss of SSTR2 was linked to metastatic progression of prostate cancers. Patients with SSTR2-negative prostate cancers had impaired metastasis-free survival, and metastases of prostate cancers expressed even lower levels of SSTR2 mRNA than primary prostate cancers. Similar findings have been described for colorectal cancers [46] and seminonas [47]. In addition, gene transfer of SSTR2 inhibited metastatic progression in two different pancreatic carcinoma models [48], [49].

How SSTR2 might regulate tumor spreading is less clear, but published data suggests a mechanism promoting cell-cell adhesions. In pancreatic cancer the Src homology region 2 domain-containing tyrosine phosphatase 1 (SHP-1) is able to de-phosphorylate the epithelial cell adhesion molecule E-cadherin, thereby stabilizing inter-epithelial cell junctions in a SSTR2-dependent fashion [48], [50]. SHP-1 has also been shown to be downregulated in biologically aggressive prostate cancers [10]. This mechanism is further supported by work of Lahlou et al. showing that SSTR2 facilitated restoration of functional gap junctions in pancreatic adenocarcinoma cells through up-regulation of connexins Cx26 and Cx43 [51].

Most interestingly, in prostate cancer cells SSTR2 is also able to inhibit another feature of cancer metastasis, cell migration [52]. Mechanistically, this seems to be mediated by ligand-dependent activation of the Y-27632-sensitive Rho-GTPase pathway [52]. Data from normal, healthy, primary keratinocytes corroborate this mechanism and suggest underlying SSTR2- (amongst others) mediated inhibition of Rac1 activity [53].

## Conclusions

We have been able to clarify that loss of SSTR2 is strongly linked to an aggressive tumor phenotype and predicts poor prognosis of prostate cancers. Indeed, most prostate cancers are SSTR2 negative and SSTR2 expression seems to be an important factor in the pathogenesis of prostate cancer. Although, the suitability of SSTR2 as a target for gene therapy needs to be evaluated, loss of SSTR2 is strongly linked to invasiveness, early PSA relapse, and metastatic spread in prostate cancer. Our data suggest that mechanistically these effects are mediated by increased cancer cell proliferation in cells with down-regulated SSTR2.
